# Supplementary material for: Who Will Save the Savior? The Relationship between Therapists’ Secondary Traumatic Stress, Secondary Stress Self-Efficacy, and Attitudes toward Trauma-Informed Care
Source: Behav Sci (Basel). 2023 Dec 13;13(12):1012. doi: 10.3390/bs13121012 (PMC10740790; doi:10.3390/bs13121012)
Supplement: Supplementary file 1 [file behavsci-13-01012-s001.zip › behavsci-2668593-supplementary.pdf]

Who Will Save the Savior? The Relationship between Therapists' Secondary Traumatic Stress, Secondary Stress Self-Efficacy, and Attitudes toward Trauma-Informed Care

Supplementary Materials

Supplementary material: Demographic and professional characteristics of the therapists

Background characteristics	Values	Frequency
Gender	Male	11 (10.9%)
	Female	90 (89.1%)
Religiousness of the therapist	Ultra – Orthodox (*Haredi*)	28 (27.7%)
	Religious	45 (44.6%)
	Traditional	5 (5.0%)
	Secular	23 (22.8%)
Profession	Social worker	74 (73.3%)
	Psychological therapist	9 (8.9%)
	Therapist through art / education professions	18 (17.8%)
Education	B. A	12 (11.9%)
	M.A	86 (85.1%)
	Ph.D.	3 (3.0%)
Intensiveness of TIC training	Without TIC training	4 (4.0%)
	Short TIC training	47 (46.5%)
	Intensive TIC training	50 (49.5%)
Initiator of the training	Without TIC training	4 (4.0%)
	Self-initiative course	47 (46.5%)
	Workplace- initiative course	50 (49.5%)
Therapy center characteristics	Local municipal service center	63 (62.4%)
	Government / national service center	17 (16.8%)
	NGO/ Independent service center	21 (20.8%)
Client's level of religiousness	Orthodox	53 (52.5%)
	Religious	13 (12.9%)
	Traditional	13 (12.9%)
	Secular	22 (21.8%)
Therapy setting	No dyad	82 (81.2%)
	Dyad treatment	19 (18.8%)
Security emergency area	no	82 (81.2%)
	yes	19 (18.8%)
TIC supervision	no	42 (41.6%)
	yes	59 (58.4%)
